# Mixed ethnicity and behavioural problems in the Millennium Cohort Study

**DOI:** 10.1136/archdischild-2015-309701

**Published:** 2016-02-24

**Authors:** Afshin Zilanawala, Amanda Sacker, Yvonne Kelly

**Affiliations:** Department of Epidemiology and Public Health, University College London, London, UK

**Keywords:** Child Psychology, Race and Health

## Abstract

**Background:**

The population of mixed ethnicity individuals in the UK is growing. Despite this demographic trend, little is known about mixed ethnicity children and their problem behaviours. We examine trajectories of behavioural problems among non-mixed and mixed ethnicity children from early to middle childhood using nationally representative cohort data in the UK.

**Methods:**

Data from 16 330 children from the Millennium Cohort Study with total difficulties scores were analysed. We estimated trajectories of behavioural problems by mixed ethnicity using growth curve models.

**Results:**

White mixed (mean total difficulties score: 8.3), Indian mixed (7.7), Pakistani mixed (8.9) and Bangladeshi mixed (7.2) children had fewer problem behaviours than their non-mixed counterparts at age 3 (9.4, 10.1, 13.1 and 11.9, respectively). White mixed, Pakistani mixed and Bangladeshi mixed children had growth trajectories in problem behaviours significantly different from that of their non-mixed counterparts.

**Conclusions:**

Using a detailed mixed ethnic classification revealed diverging trajectories between some non-mixed and mixed children across the early life course. Future studies should investigate the mechanisms, which may influence increasing behavioural problems in mixed ethnicity children.

**What is already known on this topic**

- Research has documented ethnic inequalities in health across the early life course.
- There is a paucity of longitudinal research investigating mixed ethnic differences in children's behavioural problems using a comprehensive mixed ethnic classification.

**What this study adds**

- We find White mixed, Indian mixed, Pakistani mixed and Bangladeshi mixed 3-year-old children to have fewer problem behaviours than their non-mixed counterparts.
- Growth curve models demonstrated that White mixed, Pakistani mixed and Bangladeshi mixed children experienced increases in problem behaviours compared with their non-mixed counterparts, particularly after age 7.

## Introduction

Existing evidence shows ethnic variation for a variety of markers of health across the early life course.^1–3^ Ethnic inequalities may be a consequence of group characteristics or due to social/economic disadvantage.^4^ Very little research examines the consequences of mixed ethnicity status for children.^5^ Research on mixed ethnic differences in child behaviour is limited due to broad or homogenous groups in data. Investigating children's problem behaviours during the early life course is relevant because behavioural problems have been linked to subsequent academic achievement, adult economic well-being and crime.^6^ ^7^

Only one study investigated mixed ethnic differences in behavioural problems during early childhood and reported no link between mixed ethnicity and behavioural problems among 3-year-old children.^5^ Although evidence was based on a nationally representative cohort, a single, heterogeneous ‘mixed’ category was used. A London-based study (DASH) examining 11–13-year-old children found no difference in problem behaviours between mixed black Caribbean/white and their white counterparts.^8^ Both studies use a cross-sectional design, making it difficult to discern whether and how early potential differentials in behavioural problems emerge. Another paper using DASH data looked at growth trajectories to examine problem behaviours showed more favourable child behaviour profiles among adolescent (11–16 year olds) ethnic minority groups in the UK;^9^ however, authors did not disaggregate ethnic minority groups by mixed status.

Research has called for more robust evidence on mixed ethnicity children's behavioural problems.^8^ Our analysis extends prior research in several ways. First, using a nationally representative cohort study, we estimate trajectories in behavioural problems from age 3 to 11, allowing us to detect declines or inclines in problem behaviours. Second, we use a detailed mixed ethnic classification, disaggregating ethnic groups accounts for the heterogeneity in socioeconomic, migratory and health profiles of these groups.^10^ Third, we compare the behavioural problems’ trajectories of mixed ethnicity cohort members with those of their non-mixed counterparts. This approach allows us to clarify whether children's problem behaviours are sensitive to their mixed ethnic status.

## Methods

### Data

The Millennium Cohort Study (MCS) is a nationally representative longitudinal study of 18 552 infants born in the UK between September 2000 and January 2002.^11^ The sample was clustered at the electoral ward (an administrative unit level) such that disadvantaged residential areas and areas with a high proportion of ethnic minority residents are over-represented. The main respondents are primarily mothers. The first interview was when cohort members were 9 months of age, and follow-up sweeps were conducted at ages 3, 5, 7 and 11 years. During interviews, the main respondent was asked about the cohort members’ behavioural problems.

Problem behaviours were assessed using the Strengths and Difficulties Questionnaire (SDQ),^12^ which was completed by the main respondent when cohort members were approximately 3, 5, 7 and 11 years old. We used the total difficulties score, which is the sum of four behavioural domains (peer problems, conduct disorders, hyperactivity and emotional problems) and ranges from 0 to 40 (a higher score indicates more problem behaviours). A one-point change on the SDQ is clinically meaningful.^13^ ^14^ The SDQ has been validated in ethnically diverse populations.^15^

Ethnic categories were constructed using mother's reports of the cohort child's ethnicity and were based on census categories. We drew upon father's ethnicity to facilitate the ethnic classification of the cohort child. A cohort child's ethnicity is categorised as mixed if the main respondent (usually the mother) chose a mixed category or if the ethnic categories for the child's parents were different. If the main respondent chose a ‘mixed’ ethnic category for the child, but the categories of the child’s natural parents were the same, we reclassified the child according to the parents’ ethnicity. Ethnic categories used for analysis are white; Indian; Pakistani; Bangladeshi; black Caribbean; black African; other and their ‘mixed’ counterparts. The white mixed group is made up of children whose parents are from different white backgrounds, for example, a white mixed child might have a white European mother and a white British father.

Our analyses included cohort member's gender, age, sweep at assessment as a measure of time and a quadratic term for time. The linear but not the quadratic term varied by ethnicity. We explored the impact of equivalised household income in quintiles, measured as a time-varying variable (middle-income quintile is reference)^16^ in sensitivity analyses.

Behavioural outcomes are moderated by multiple births,^17^ and therefore, we analysed data on singleton born children. We excluded from our analysis children who were reported to have attention-deficit/hyperactivity disorder, autism or Asperger's syndrome. The sample was restricted to children for whom a parent report of problem behaviours was available and for whom ethnicity was observed. For descriptive results, we estimated means on problem behaviours using cross-sectional samples. For the growth curve analyses, we pooled data from ages 3, 5, 7 and 11, allowing each child to contribute more than one observation. The pooled analyses had 16 330 children and a total of 51 509 child-years.

### Statistical analysis

We estimated growth curve models for children's behavioural problems trajectories for the analytic sample.^18^ We estimated a behavioural problems intercept and slope for each cohort member across time (level 1), regressed the mean intercept and slope on each cohort member's ethnicity (level 2), and estimated the residual variance in the intercept and slope. We present figures illustrating initial problem behaviours at age 3 and trajectories in problem behaviours by non-mixed and mixed ethnic groups. All analyses are weighted to adjust for non-response and the unequal probability of being sampled.

## Results

### Mixed ethnicity and children's problem behaviours

Table 1 presents mean problem behaviours by non-mixed and mixed ethnicity across the early life course. All sample sizes are reported in online supplementary appendix table 2. At age 3, most mixed ethnicity children had fewer problem behaviours compared with their non-mixed counterparts; White mixed, Indian mixed, Pakistani mixed and Bangladeshi mixed children had fewer problem behaviours than their non-mixed counterparts. Black Caribbean non-mixed and mixed children did not differ in their mean scores. Black African mixed children had slightly more problem behaviours compared with their non-mixed counterparts, but means were not statistically different (9.5 vs 9.2, respectively).

10.1136/archdischild-2015-309701.supp1 Supplementary tables

**Table 1:** Problem behaviours by non-mixed and mixed ethnicity at ages 3, 5, 7 and 11

Age	White non-mixed	White mixed	Indian non-mixed	Indian mixed	Pakistani non-mixed	Pakistani mixed	Bangladeshi non-mixed	Bangladeshi mixed	Black Caribbean non-mixed	Black Caribbean mixed	Black African non-mixed	Black African mixed	Other non-mixed	Other mixed
3	9.4	8.3	10.1	7.7	13.1	8.9	11.9	7.2	10.6	10.6	9.2	9.5	10.5	9.6
5	7.0	5.9	7.3	6.1	9.9	7.9	8.7	6.3	9.0	8.0	7.0	7.8	7.5	7.8
7	7.2	6.4	7.6	5.7	9.7	9.3	8.8	7.4	8.8	9.2	6.6	8.0	7.3	6.9
11	7.7	6.9	7.5	6.3	8.8	10.3	7.8	12.0	7.9	8.7	7.4	6.7	8.4	7.8

All means are adjusted for sample design. Sample at each sweep excludes twins and triplets. Only respondents who are biological, step, adopted or foster mothers are included. At ages 5, 7 and 11, children who had attention-deficit/hyperactivity disorder/Asperger’s or autism are excluded. The sample size at age 3 is 13 742; age 5 is 13 555; age 7 is 12 250; and age 11 is 11 996.

The patterns in problem behaviours are less consistent at age 11. White and Indian mixed children have fewer problem behaviours compared with their non-mixed counterparts. Pakistani, Bangladeshi and Black Caribbean mixed children have more problem behaviours than their non-mixed counterparts. Black African mixed children have fewer behavioural problems than their non-mixed counterparts. In order to closely scrutinise the increase and decrease in problem behaviours over time, we turn to growth curve models to more accurately distinguish differences between mixed ethnicity children and their counterparts.

### Growth trajectories

**Figure 1:**
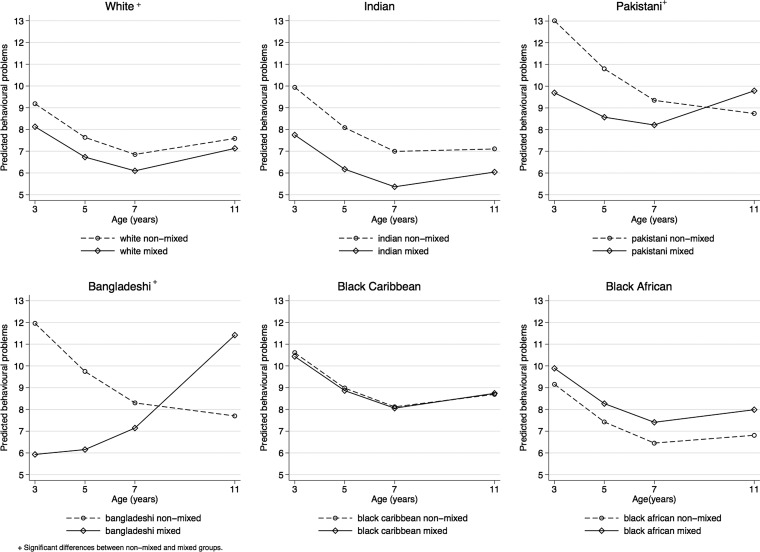
Predicted behavioural problems by mixed ethnicity.

Figure 1 displays the average fitted growth trajectories in problem behaviours by mixed status conditional on age and gender. Regression coefficients are in online supplementary appendix table 1 (model 1). The rate of growth in problem behaviours for white mixed children is significantly different from their non-mixed counterparts (b=0.075, se=0.037). Indian children experienced a decline in problem behaviours over time and trajectories do not differ between non-mixed and mixed Indian children. Pakistani and Bangladeshi mixed children experience rates of change in problem behaviours that are significantly different from those of their non-mixed counterparts. There were no differences in the rates of change between non-mixed and mixed black Caribbean children. Lastly, black African non-mixed children experienced a decline in problem behaviours but their trajectories did not significantly differ from their mixed counterparts.

In sensitivity analyses, adjustments for equivalised household income decreased the mean differences in intercepts of child behaviour between mixed and non-mixed ethnic minorities. However, the magnitude and significance of trajectories were not sensitive to adjustments for income (see online supplementary appendix table 1, model 2).

## Discussion

In this paper, we extend our understanding of mixed ethnicity differences in behavioural problems by examining children's trajectories beginning in early childhood and including a comprehensive mixed ethnic classification using nationally representative data. At age 3, we find white mixed, Indian mixed, Pakistani mixed and Bangladeshi mixed children to have fewer problem behaviours than their non-mixed counterparts. This finding is in stark contrast to previous research finding no differences in problem behaviours between a catch-all mixed group and non-mixed 3-year-old children.^5^ Growth curve models show that white mixed, Pakistani mixed and Bangladeshi mixed children experienced increases in problem behaviours compared with their non-mixed counterparts, most notably after age 7.

Our findings suggest that by age 11 some mixed children have more problem behaviours than their non-mixed counterparts. There are two possible explanations for this result. One, mixed ethnicity as a category may reflect not only social stratification processes but also shifts in identity and potential confusions between personal and social identities.^19^ The behavioural problems among mixed 11-year-old children in our data may very well reflect the strain from the nuanced nature of forming and maintaining a mixed ethnicity heritage. Second, we do not observe differences in problem behaviours between non-mixed and mixed black Caribbean children, but such differences are evident between non-mixed and mixed statuses among Pakistani and Bangladeshi children. This could be explained by the normative nature of interethnic partnerships among the black Caribbean community, while mixed partnerships are less common in South Asian communities.^5^ It could be that mixed partnerships yield less anxiety and strain, factors affecting child outcomes, when such interethnic families are more common within an ethnic group. Future studies should disentangle the influence of family context, psychosocial factors and problem behaviours among mixed ethnicity children.

The aim of this short report was to describe, for the first time, trajectories in problem behaviours for non-mixed and mixed ethnicity children in a nationally representative sample. White mixed, Pakistani mixed and Bangladeshi mixed children had significantly different growth trajectories compared with their non-mixed counterparts. Future research might examine the mechanisms, such as school, psychosocial and socio-demographic factors, behind these differences.
