# Supplementary material for: Mixed ethnicity and behavioural problems in the Millennium Cohort Study
Source: Arch Dis Child. 2016 Feb 24;103(1):61–4. doi: 10.1136/archdischild-2015-309701 (PMC5754876; doi:10.1136/archdischild-2015-309701)
Supplement: Supplementary tables [file archdischild-2015-309701supp001.pdf]

Appendix Table 1. Growth curve analyses predicting behavioural problems

	**Model 1**	**Model 2**
Wave	-0.96***	-0.97***
	(0.021)	(0.021)
Wave squared	0.096***	0.097***
	(0.0024)	(0.0024)
Child's age	-0.70***	-0.70***
	(0.098)	(0.098)
Child is male	1.03***	1.03***
	(0.081)	(0.077)
White mixed	-1.06***	-0.80***
	(0.21)	(0.21)
Indian non-mixed	0.71**	0.72**
	(0.33)	(0.33)
Indian mixed	-1.48***	-1.20**
	(0.57)	(0.56)
Pakistani non-mixed	3.86***	3.17***
	(0.29)	(0.28)
Pakistani mixed	0.54	0.023
	(0.80)	(0.75)
Bangladeshi non-mixed	2.81***	2.10***
	(0.46)	(0.46)
Bangladeshi mixed	-3.22	-3.00
	(2.12)	(1.87)
Black Caribbean non-mixed	1.42***	0.95**
	(0.41)	(0.40)
Black Caribbean mixed	1.25***	0.92**
	(0.44)	(0.42)
Black African non-mixed	-0.031	-0.41
	(0.35)	(0.34)
Black African mixed	0.70	0.51
	(0.69)	(0.63)
Other non-mixed	1.13**	0.86*
	(0.50)	(0.49)
Other mixed	0.32	0.25
	(0.41)	(0.41)
White mixed * wave	0.075**[a]	0.080**[a]
	(0.037)	(0.037)
Indian non-mixed * wave	-0.15***	-0.17***
	(0.051)	(0.051)
Indian mixed * wave	-0.014	-0.017

	(0.092)	(0.093)
Pakistani non-mixed * wave	-0.33***[a]	-0.37***[a]
	(0.045)	(0.046)
Pakistani mixed * wave	0.21*	0.22*
	(0.13)	(0.13)
Bangladeshi non-mixed * wave	-0.33***[a]	-0.37***[a]
	(0.070)	(0.071)
Bangladeshi mixed * wave	0.89	0.81
	(0.59)	(0.57)
Black Caribbean non-mixed * wave	-0.040	-0.036
	(0.088)	(0.088)
Black Caribbean mixed * wave	-0.012	-0.00013
	(0.063)	(0.063)
Black African non-mixed * wave	-0.094*	-0.10*
	(0.052)	(0.053)
Black African mixed * wave	-0.038	-0.047
	(0.10)	(0.10)
Other non-mixed * wave	-0.071	-0.075
	(0.086)	(0.086)
Other mixed * wave	-0.067	-0.048
	(0.069)	(0.070)
Lowest income quintile		1.14***
		(0.084)
Second income quintile		0.64***
		(0.070)
Fourth income quintile		-0.47***
		(0.061)
Top income quintile		-0.87***
		(0.068)
Constant	8.66***	8.68***
	(0.065)	(0.075)
Child-years	51,509	51,509
Number of children	16,330	16,330

Robust standard errors in parentheses

*** p<0.01, ** p<0.05, * p<0.10

[a]Significant differences between non-mixed and mixed groups within ethnic group.

Appendix Table 2. Mixed ethnicity frequencies by age

	Age 3	Age 5	Age 7	Age 11
White non-mixed	11420	11253	10116	9729
White mixed	449	437	389	365
Indian non-mixed	290	298	270	261
Indian mixed	52	51	54	53
Pakistani non-mixed	444	445	426	509
Pakistani mixed	34	43	36	39
Bangladeshi non-mixed	152	143	149	219
Bangladeshi mixed	11	10	9	12
Black Caribbean non-mixed	149	140	128	115
Black Caribbean mixed	180	171	147	144
Black African non-mixed	200	215	201	203
Black African mixed	66	59	52	57
Other non-mixed	143	126	130	149
Other mixed	152	165	143	141
Total	13742	13556	12250	11996

Notes: At each age, sample size is conditional on observed ethnicity and child behaviour and excludes multiple births and children who had ADHD/Asperger's syndrome or Autism.
